# New clinical application prospects of artemisinin and its derivatives: a scoping review

**DOI:** 10.1186/s40249-023-01152-6

**Published:** 2023-12-11

**Authors:** Yangmu Huang, Yang Yang, Guangqi Liu, Ming Xu

**Affiliations:** 1 School of Public Health, Peking University, 38 Xue Yuan Road, Haidian District, Beijing, 100191 China; 2 Institute for Global Health and Development, Peking University, 38 Xue Yuan Road, Haidian District, Beijing, 100191 China; 3 Energy Saving and Environmental Protection and Occupational Safety and Health Research Institute, China Academy of Railway Sciences Co., Ltd, No. 2 Daliushu Road, Beijing, 100081 China

**Keywords:** Artemisinin, Derivatives, Medicinal effect, Clinical application, Clinical study, Scoping review

## Abstract

**Background:**

Recent research has suggested that artemisinin and its derivatives may have therapeutic effects on parasites, viruses, tumors, inflammation and skin diseases. This study aimed to review clinical research on artemisinin and its derivatives except anti-malaria and explore possible priority areas for future development.

**Methods:**

Relevant articles in English and Chinese published before 28 October 2021 were reviewed. All articles were retrieved and obtained from databases including WanFang, PubMed/MEDLINE, the Cochrane Library, China National Knowledge International, Embase, OpenGrey, the Grey Literature Report, Grey Horizon, and ClinicalTrials.gov. Studies were selected for final inclusion based on predefined criteria. Information was then extracted and analyzed by region, disease, outcome, and time to identify relevant knowledge gaps.

**Results:**

Seventy-seven studies on anti-parasitic (35), anti-tumor (16), anti-inflammatory (12), anti-viral (8), and dermatological treatments (7) focused on the safety and efficacy of artemisinin and its derivatives. The anti-parasitic clinical research developed rapidly, with a large number of trials, rapid clinical progress, and multiple research topics. In contrast, anti-viral research was limited and mainly stayed in phase I clinical trials (37.50%). Most of the studies were conducted in Asia (60%), followed by Africa (27%), Europe (8%), and the Americas (5%). Anti-parasite and anti-inflammatory research were mainly distributed in less developed continents such as Asia and Africa, while cutting-edge research such as anti-tumor has attracted more attention in Europe and the United States. At the safety level, 58 articles mentioned the adverse reactions of artemisinin and its derivatives, with only one study showing a Grade 3 adverse event, while the other studies did not show any related adverse reactions or required discontinuation. Most studies have discovered therapeutic effects of artemisinin or its derivatives on anti-parasitic (27), anti-tumor (9), anti-inflammatory (9) and dermatological treatment (6). However, the efficacy of artemisinin-based combination therapies (ACTs) for parasitic diseases (non-malaria) is still controversial.

**Conclusions:**

Recent clinical studies suggest that artemisinin and its derivatives may be safe and effective candidates for anti-tumor, anti-parasitic, anti-inflammatory and dermatological drugs. More phase II/III clinical trials of artemisinin and its derivatives on antiviral effects are needed.

**Graphical Abstract:**

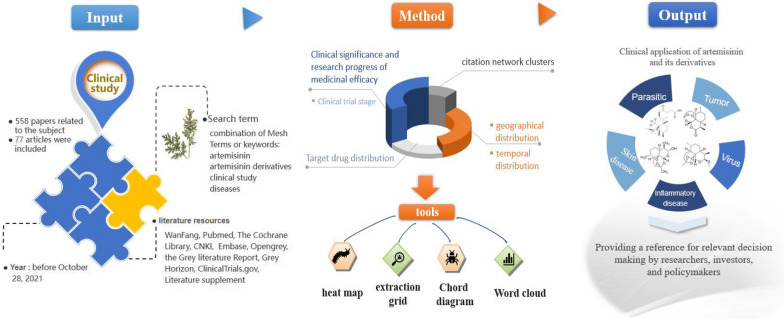

**Supplementary Information:**

The online version contains supplementary material available at 10.1186/s40249-023-01152-6.

## Background

Artemisinin is a natural sesquiterpene lactone obtained from *Artemisia annua* herb, which has been confirmed to be effective in the treatment of different forms of malarial parasites and thus has attracted much attention [1, 2]. Since the 1980s, artemisinin derivatives have gradually become the focus of research due to their low cost, good effect and ease of use [3–5]. In 2006, artemisinin-based combination therapies (ACTs) were recommended by the World Health Organization (WHO) as first-line treatment of falciparum malaria [6, 7]. At present, artemisinin and its derivatives have become the most important and effective antimalarial drugs [8]. However, the appearance of drug-resistant *Plasmodium falciparum* promotes the continuous improvement of antimalarial drug research and development, such as artemisinin-based combination therapies [8–10].

Early studies of artemisinin and its derivatives mainly focused on antimalarial effects. However, the low toxicity and immune regulation of artemisinin and its derivatives in the process of malaria treatment have lighted the interest of researchers in many other disease fields [11–15]. Over the past few decades, the therapeutic effects of artemisinin and its derivatives on various diseases have been discovered through a large amount of in vivo and in vitro studies, and have gradually led to the increase of clinical trials [16]. The possible effect of artemisinin and its derivatives for the treatment of Corona Virus Disease 2019 (COVID-19) has again attracted research interest.

To our knowledge, the results of these clinical studies have not yet been summarized. Thus, this scoping review aimed to overview the current status of clinical research on artemisinin and its derivatives on anti-parasite (non-malaria), antivirus, anti-tumor, inflammatory and dermatosis therapy and hope to explore potential priority areas for future development.

## Methods

### Inclusion and exclusion criteria

According to the review’s purpose, we conducted a scoping review guided by Arksey and O’Malley’s methodological framework [17]. We included all articles on the clinical use of artemisinin and its derivatives published before October 28, 2021. The following types of articles were excluded: studies on the antimalarial effects of artemisinin and its derivatives, conference papers, articles without research data, articles that did not address human diseases, duplicate publications, and studies that were not conducted based on clinical trials.

### Retrieval and search method

Two authors searched for articles in the following databases: Wanfang (wanfangdata.com.cn), CNKI (cnki.net), PubMed/MEDLINE, the Cochrane Library, Embase, OpenGrey, the Grey Literature Report, Grey Horizon, and ClinicalTrials.gov. The search strategy was defined for each database by using a combination of MeSH terms or keywords including artemisinin, artemisinin derivatives, artesunate, dihydroartemisinin, artemether, clinical study, and diseases. The keywords were adapted for each database to be consistent with their indexing. The literature search was conducted on October 28, 2021. Mendeley 1.19.5 (Elsevier, Amsterdam, NL) and Endnote20 (Clarivate Analytics, Pennsylvania, US) software were used to manage references and remove duplicates.

### Study selection

We performed a pilot selection study to evaluate consistency in the application of the above criteria and to identify discrepancies with 20 randomly selected references. For both abstract and full-text screening, two independent reviewers selected studies by title and abstract/full text, and the third reviewer resolved disagreements. Eligible clinical trials were screened by two independent reviewers (YY and GL) by title and abstract/full text based on study subjects, study diseases, study type, and details. In case of disagreement, a third reviewer (YH) was invited to review until a consensus was reached.

### Data extraction, summary, and analysis

An extraction grid was created to record the following information for each of the selected studies: title, author, time, drug, disease, scale, safety, results, country, viewpoint, and conclusion. Initially, the three contributors extracted data from the same 10 articles independently to ensure harmonization. The other two participants resolved disagreements in the discussion. Subsequently, the remaining 67 articles were then summarized, their quality assessed by the same three contributors, and the results were recorded in the extraction grid (Additional file 1: Table S1). A quantitative conventional content analysis was employed to summarize and report the results. The key findings were classified into specific categories derived from the articles rather than a predefined framework. The categorization was revised with the advice of the panel of experts. Priority was given to the classification of the study itself. The remaining studies were classified into viral, parasitic, tumor, dermatologic, and inflammatory diseases using the International Classification of Diseases, 11th Revision (ICD-11) in combination with the disease classification of previous studies. As an emerging disease, COVID-19 was classified according to the primary outcome of the study.

## Results

### Description and general characteristics of the included studies

**Fig. 1:**
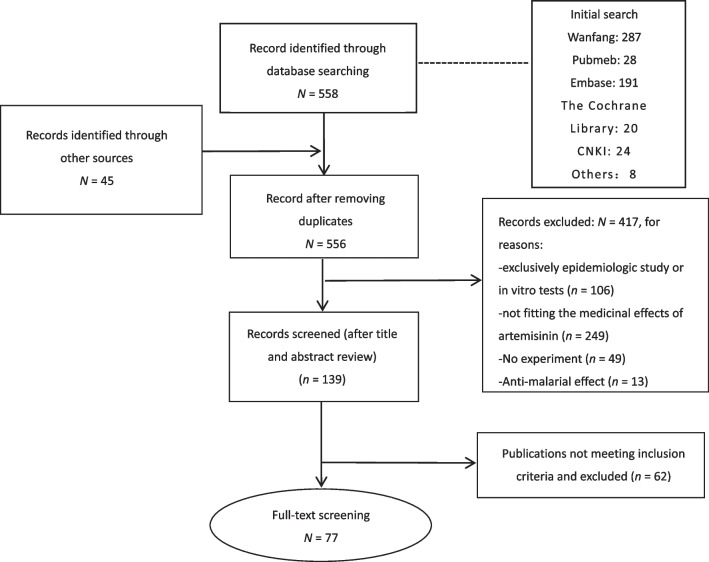
Flowchart of literature screening and selection process

**Fig. 2:**
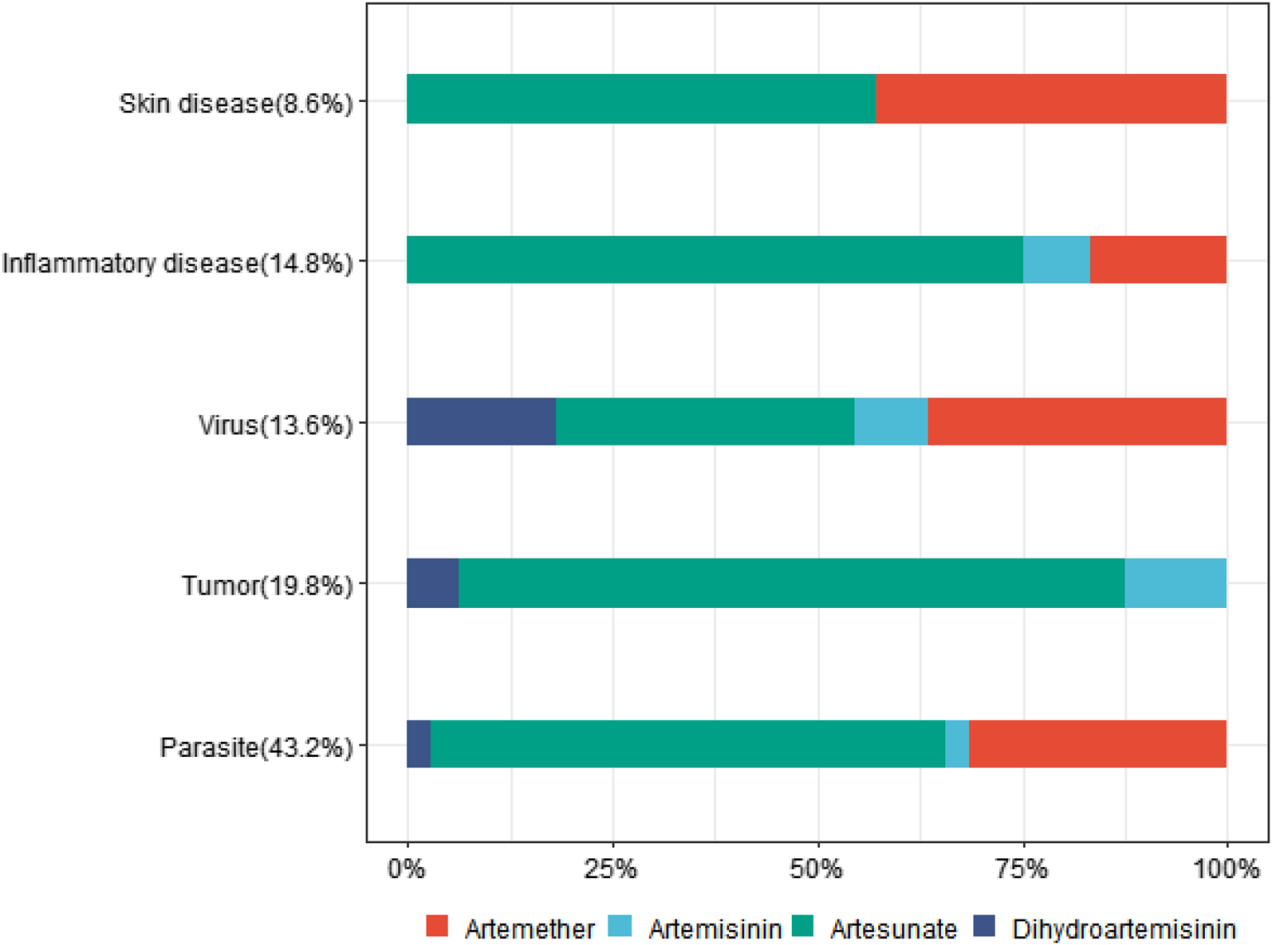
Percentage of clinical studies of different artemisinin and its derivatives classified by disease field

Our study found 558 articles after matching the keywords. By title and abstract screening, 62 articles were excluded according to our inclusion and exclusion criteria. According to the inclusion and exclusion criteria, 77 articles were finally included after more detailed full-text assessment and reference review. The article search and selection processes are shown in Fig. 1. The diseases, target drugs and research time of this study are shown in Table 1 and Fig. 2.

**Table 1:** General characteristics of the included studies (one study included more than one study drug)

	Artemisinin	Artesunate	Dihydroartemisinin	Artemether	Total	%
*Drug purpose*						
Parasite	1	22	1	11	35	43.2
Tumor	2	13	1	0	16	19.8
Virus	1	4	2	4	11	13.6
Inflammatory disease	1	9	0	2	12	14.8
Skin disease	0	4	0	3	7	8.6
*Year of publication*						
Before 2000	0	9	0	2	11	13.8
In or after 2000	5	42	4	18	69	86.2

Clinical research on artemisinin and its derivatives (except malaria treatment) began in the 1990s, and the number of studies has gradually increased (Additional file 1: Table S1). The main study subjects were artesunate and artemether (Fig. 2). Of the 77 retained studies, most were conducted in Asia (60%), followed by Africa (27%), Europe (8%), and the Americas (5%) (Table 2). Adverse events in all the included studies were graded according to the National Cancer Institute criteria, ranging from grade 1 (no events) to grade 5 (life-threatening events). Of the 59 studies with documented adverse events, only one reported a grade 3 adverse event without drug discontinuation, and all the rest 58 studies did not show any related adverse effects.

**Table 2:** Final selection of 77 references on group of diseases by continent

Continent	Parasite	Tumor	Inflammatory disease	Virus	Skin disease	Total	%
Asia	21	8	10	2	6	47	60
Australia	0	0	0	0	0	0	0
Africa	13	0	2	5	1	21	27
Europe	0	5	0	1	0	6	8
Americas	1	3	0	0	0	4	5

**Fig. 3:**
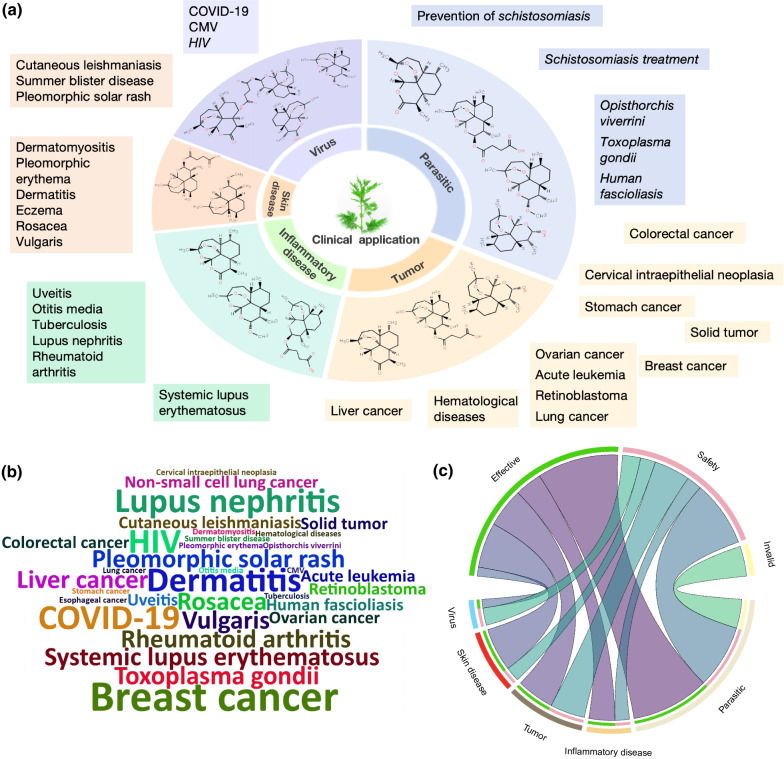
**a** Distribution of potential clinical applications of artemisinin and its derivatives. **b** Word cloud of research results on efficacy/safety for diseases (due to the excessive number of studies on schistosomiasis control, the image is not shown in this figure). The graph was based on the frequency of clinical studies of artemisinin and its derivatives in the treatment of different diseases. **c** Research status on the efficacy and safety of artemisinin and its derivatives for potential clinical applications. The upper semicircle represents the research results, and the lower semicircle represents the field of disease. Different colors are used to distinguish the research results, that is, safe in pink, effective in green and invalid in yellow. The width of the string is based on the number of studies that have corresponding results in this field

As for different disease types, studies on tumors are the most, covering 12 different diseases, such as breast cancer, liver cancer, ovarian cancer. In addition, studies on dermatology cover 10 different diseases, such as rosacea, eczema, and dermatitis (Fig. 3a). The number of clinical research on schistosomiasis, breast cancer, COVID-19, Human immunodeficiency virus (HIV) and dermatitis are relatively high (Fig. 3b). According to the Chord diagram (Fig. 3c), the effectiveness of artemisinin and its derivatives in the treatment of parasitosis, skin diseases, inflammatory diseases and tumors have received more attention, while the antiviral research still mainly focused on safety.

### Antiparasitic effect

Artemisinin and its derivatives are known to be one of the most effective antimalarial drugs. Their use in the treatment of malaria has reduced the number of malaria-related cases and deaths worldwide by 30% and 37%, respectively, and reduced the number of deaths among children under 5 years of age by 11% as of 2019 [8]. In addition to their antimalarial effect, artemisinin and its derivatives also showed effects on other parasites, such as *Schistosoma japonicum*, *Fasciola* and *Toxoplasma gondii* [18, 19].

#### Anti-schistosomiasis effect

Clinical studies have suggested that artemisinin and its derivatives showed significant anti-*S. japonicum* properties, and were safe and cost-effective [20, 21]. Related research has entered phase III clinical trials. In terms of safety, repeated oral administration of artemisinin and its derivatives did not cause any related adverse reactions in phase I clinical trial. A single dose of 6 mg/kg of artemether, artesunate or artemisinin has been proven to be safe in many studies [22]. The study by Hua et al. suggested that this dose of artesunate was appropriate to be taken once a week for 4 weeks [23]. In terms of efficacy, most studies have suggested that artemether is effective in the early treatment of acute schistosomiasis, and can reduce the infection rate and intensity of the disease [20, 21, 24–27]. However, in some studies, the efficacy of artemisinin derivatives alone is not significant in clinical trials [28].

Artemisinin and its derivatives are also proven to be effective in schistosomiasis prevention. Lin et al. proved that taking a single dose of 6 mg/kg every 15 days has a preventive effect [29]. Other follow-up studies also supported that artemisinin and its derivatives have obvious preventive effects on residents in schistosomiasis-endemic areas [22, 25, 26, 29–35]. Research suggested that artemisinin derivatives had a wide range of prevention and protection rates, for example, the rates for artesunate reached 60.8–100% [29–34, 36, 37], and the rate for artemether reached 25–95% [25, 26, 35, 38]. It is noteworthy that the sensitivity of *S. japonicum* to artesunate decreased after its long-term use [39–41].

Artemisinin-based combination therapies (ACTs) may be used as an adjuvant measure for schistosomiasis treatment, but this approach is highly controversial. Researchers agreed that artemisinin-based combination therapies were safe and well tolerated, similar to praziquantel, the current preferred treatment for schistosomiasis [42, 43]. Some clinical studies found that ACTs can specifically reduce transmission and help eliminate schistosomiasis [44]. Their use in the treatment of urinary schistosomiasis [45] and intestinal schistosomiasis [46] were safe and effective. Notably, the curative rate was not significantly different from that of praziquantel alone [47], and even an open randomized controlled trial in western Kenya found that standard praziquantel therapy was more effective. The role of ACT for schistosomiasis needs to be further studied [48].

#### Anti-*Toxoplasma gondii* effect

Existing studies suggested that artemisinin and its derivatives are safe in the treatment of toxoplasmosis, but it is uncertain whether they can alleviate schizophrenia caused by *T. gondii* infection. In terms of safety, phase I clinical trials have shown that artemisinin and its derivatives are safe when use alone or in combination with antipsychotics. However, their efficacy in the treatment of toxoplasmosis is still controversial. Wang et al. [43] also confirmed that artemether could significantly improve some mental symptoms caused by *T. gondii* infection, and that combination with antipsychotics could effectively reduce the positivity rate of *T. gondii* antibodies. However, some studies have shown that this combination therapy did not improve clinical symptoms such as cognitive impairment in patients with schizophrenia [49, 50].

#### Anti-trematodes effect

Studies have suggested that artemisinin and its derivatives may also have some inhibitory effect on *Fasciola hepatica* [51], but there is a lack of research on the effect of ACTs [52]. A randomized controlled pilot study of human fascioliasis in central Vietnam found that artemisinin and its derivatives were effective against fascioliasis [51]. A subsequent clinical trial confirmed this result but found that the therapeutic effect was not sufficient to replace triclabendazole, the standard anti-fascioliasis drug. The role of artemisinin derivatives as matching drugs in combination therapies is not clear [52].

### Study on the antitumor effect of artemisinin and its derivatives

Clinical studies have suggested that artemisinin and its derivatives are safe for tumor treatment, and might have effects on different kinds of tumors, including liver cancer, ovarian cancer, breast cancer, non-small cell lung cancer, colorectal cancer, cervical intraepithelial neoplasia, retinoblastoma, etc. [53–60]. A phase I clinical trial found that the apparent clearance rate increased with time after artesunate use in patients with breast cancer [61]. The Maximum tolerated dose (MTD) for intravenous injection was 18 mg/kg. At this dose, adverse reactions were mild and self-limited [62]. Under the clinically effective dose for the treatment of Cervical Intraepithelial Neoplasia (CIN)2/3, transvaginal injection of artesunate is safe and well tolerated [60]. Oral ART doses up to 200 mg per day are safe and well tolerable in patients with metastatic breast cancer [59, 63]. The recommended dose for phase II/III clinical trials is 200 mg/day [58]. Clinical studies have suggested that artemisinin and its derivatives might have a better clinical effect in tumor treatment by inhibiting tumor angiogenesis [64] and effectively improving immunity in patients with primary liver cancer and hematological diseases [54, 65]. In addition, a study found that hepatic arterial infusion of artesunate was similar to conventional Transcatheter arterial chemoembolization (TACE) therapy in reducing tumor size, reducing the short-term efficacy of AFP, and reducing interventional side effects [54].

Artesunate combined with anti-tumor drug therapy was also safe and effective in ovarian cancer and non-small cell lung cancer [55]. In the treatment of ovarian cancer, artesunate increases sensitivity to cisplatin [55, 66]. Artesunate combined with NP and chemotherapy combined with sequential administration of artesunate can improve the disease control rate [55, 67].

### Treatment of inflammatory diseases

Artemisinin and its derivatives have therapeutic effects on some infectious inflammatory diseases [68–70] and immune inflammatory diseases [71–76]. At present, few clinical trials have been conducted on the anti-inflammatory effects of artemisinin and its derivatives. Long-term clinical studies on artemisinin and its derivatives in the treatment of articular systemic lesions of Lupus nephritis (LN) [73, 76], Vogt-Koyanagi-Harada (VKH) syndrome [68, 77] and systemic lupus erythematosus suggested their safety with no obvious side effects [78]. It is worth noting that the pharmacokinetic parameters of artemether decreased significantly in tuberculosis treatment by rifampicin. This suggested that artemether should not be used in combination with rifampicin [79]. Other clinical studies have suggested that artemisinin derivatives are effective in the treatment of rheumatoid arthritis, joint systemic lesions of LN, VKH syndrome, COVID-19 and chronic simple otitis media [68–70, 74, 75]. In particular, the curative effect on rheumatoid arthritis was no less than that of the commonly used drug hydroxychloroquine [74, 75]. In addition, artemisinin could also inhibit the recurrence of lupus nephritis [71, 72, 80], further broadening its clinical application.

### Antiviral effects of artemisinin and its derivatives

Research on the antiviral effects of artemisinin and its derivatives has mostly remained at the basic research stage, mainly in phase I clinical trials, and few clinical trials have been carried out. The phase I clinical trials showed that the combination with lopinavir and ritonavir significantly increased the exposure to clomiphene and decreased the exposure to artemether [81, 82]. It is predicted that 240 mg of artemether can achieve therapeutic plasma artemether concentrations [83]. In the case of the recommended dose of artemether-clomiphene, enhanced monitoring did not raise any safety concerns [82]. These results support for the continuation of phase II clinical trials. A prospective study on COVID-19 patients found that artemisinin combined with other drugs could clear pathogens from COVID-19 patients, shorten treatment time and improve prognosis [70]. However, another clinical study found that the standard 3-day artemisinin antimalarial regimen (4 mg/kg/day for 3 days) had no detectable effect on Cytomegalovirus (CMV) viremia in children with malaria. It is speculated that effective treatment of cytomegalovirus infection may require a longer course and/or a higher dose of artesunate than for conventional malaria [84].

### Treatment of skin diseases

In clinical applications, artemisinin and its derivatives were found to be safe in the treatment of connective tissue diseases such as dermatitis (eczema), photosensitive dermatosis, summer blister disease, psoriasis vulgaris, and dermatomyositis [85–90]. Studies have found that artemisinin and its derivatives are effective [85] in treating some skin diseases. For example, the effective rate of artesunate in the treatment of eczema was 100%, and the effective rate in the treatment of pleomorphic erythema, pleomorphic solar eruption and summer blisters were 100% [86]. The effective rates for the treatment of psoriasis vulgaris and dermatomyositis were 60% and 75%, respectively [86]. In addition, artemisinin and its derivatives appeared to have good long-term efficacy in the treatment of skin diseases, such as eczema and photosensitive dermatoses with artemisinin [85], and rosacea with artesunate [88]. However, their efficacy in treating atopic dermatitis appeared to be poor [85]. Artemisinin and its derivatives appeared to have an advantage over some common dermatological drugs in the treatment of mild to moderate skin diseases. For rosacea, artesunate is similar in efficacy to doxycycline [87] and improves the condition earlier than metronidazole [88]. For acne vulgaris, artemether is more effective than fusidic acid [90].

## Discussion

The purpose of this scoping review was to overview the current situation of the clinical application of artemisinin and its derivatives, and to seek how to maximize the utility of artemisinin within a range of therapeutic effects [91]. The main study subjects were artesunate and artemether. Even though research on the treatment of inflammatory diseases, tumors and skin diseases were developing relatively fast, the research related to anti-parasites were the most advanced in terms of clinical progress and quantity. The safety of artemisinin and its derivatives have been proven in most clinical trials [92], but the therapeutic effects vary in different disease areas. From the perspective of geographical distribution, studies on anti-parasitic and anti-inflammation were mainly in Asia, Africa and other developing areas, whereas the developed countries paid more attention to anti-tumor research. In addition, studies suggested that artemisinin and its derivatives might be more cost-effective compared with the existing standard treatments in various disease fields [5, 93–99]. In general, artemisinin and its derivatives have certain development prospects in the fields of anti-parasite, anti-virus, anti-inflammatory diseases, anti-tumor and skin diseases.

For parasites, the effect of artemisinin or its derivatives has been demonstrated, especially for artesunate and artemether. However, the efficacy of ACTs is still controversial [46, 48]. Some studies suggest that the occurrence of parasitic diseases can be explained by environmental epidemiology [100], and stratified sampling should be carried out in clinical research according to regional characteristics. It is worth noting that artemisinin and its derivatives are currently the most widely used antimalarial drugs, and widespread used in the treatment of other parasitic diseases may increase the risk of drug resistance in malaria parasites. Whether artesunate can be widely used to treat schistosomiasis in malaria-endemic areas requires further consideration and long-term monitoring [101–103].

For tumors, the clinical research of artemisinin and its derivatives was relatively mature, involving a wide range of diseases and their curative effects. However, their antitumor effect does not show absolute advantages compared with standard antitumor drugs [54, 65]. But with the emergence of drug resistance to standard anti-tumor drugs, it is imperative to explore new anti-tumor drugs with excellent efficacy and few side effects [104]. Related research should focus on exploring the tumor inhibition capabilities of artemisinin and its derivatives for further development, and focus on exploring the synergistic and sensitizing effects of them with standard antitumor treatments [105, 106].

For inflammation, compared with standard inflammatory treatments, artemisinin and its derivatives could significantly improve the curative effect, shorten the course of the disease, and reduce recurrence and complications, which is worth popularizing in public hospitals [69]. However, it is suggested that artemisinin and its derivatives might interact with some standard inflammatory drugs [79]. Therefore, attention should be given to drug-drug interactions. At the same time, more randomized clinical trials should be conducted to translate the plethora of preclinical results into clinical practice.

For skin diseases, artemisinin and its derivatives seem to be more effective than some dermatological drugs; in the treatment of papulopustular rosacea, the disease improved earlier and the beneficial effect lasted longer [86]. Currently, topical or oral corticosteroids are often used to treat skin diseases, which can easily cause various adverse reactions [107], but artemisinin and its derivatives are rare. Therefore, in terms of cost, safety and efficacy, artemisinin and its derivatives might have more advantages than some dermatological drugs in the treatment of skin diseases. More clinical trials are recommended to explore their mechanism of action and therapeutic potential [101, 108].

For viruses, artemisinin and its derivatives may also have broad application prospects, particularly in the control of COVID-19 [109, 110]. However, relevant clinical studies are still in the exploratory stage, and lack serious phase I clinical trials. Studies have suggested that artemisinin and its derivatives have an inhibitory effect on novel coronavirus [70], and might have the potential to treat COVID-19 [111, 112]. In addition, in the study of the antiviral effects of artemisinin and its derivatives, the results of some clinical trials might be contrary to the results of basic research and animal trials, such as cytomegalovirus. This might be due to improper course of treatment or dose design [84]. Therefore, attention should be paid to the structure-tissue exposure/selectivity relationship, that is, the delicate balance between clinical dose/efficacy/toxicity [113]. At the same time, the scope of randomized clinical trials should be expanded based on the basic research results.

Artemisinin and its derivatives represent a new beginning for antimalarial treatment worldwide, and great efforts have been made for their pharmacological study and mechanism exploration [114]. As a "modern traditional Chinese medicine", artemisinin provided a model for the development of traditional medicine using modern science and technology [115, 116]. Traditional medicine might be more suitable for the research and development of pharmaceutical products related to parasitic and other complex diseases [115, 116].

Clinical studies on malaria treatment with artemisinin and its derivatives are relatively mature and have been conducted on a large scale. Therefore, malaria-related studies were excluded from the literature search to limit the number of studies. Additionally, our included studies themselves might have some limitations to influence our conclusion. For example, the sample sizes for some studies were too small, so the results might have been due to chance; while some studies did not report the sampling strategies. Clinical trials of antiparasitic effects are mainly conducted in China, and there are almost no co-epidemic areas of schistosomiasis japonica and falciparum malaria. Thus, risk for drug resistance may not be taken into account [117]. In addition, most of the included studies involved randomized controlled trials (44, 57.1%), followed by quasi-experiment studies (28, 36.4%), and others (5, 6.5%). The overall quality of the included studies was good, but the quality of other types of evidence was relatively general, which may affect the credibility of the results to some extent.

## Conclusions

Except antimalarial effects, current clinical research suggested that artemisinin and its derivatives might be safe and effective on anti-tumor, anti-virus, anti-parasite, anti-inflammatory and skin disease treatment. The potential to treat inflammatory diseases may provide a promising candidate to treat and suppress the recurrence of inflammatory and autoimmune diseases, and could potentially be an option for the urgent treatment for the COVID-19 pandemic. However, the underlying mechanism is not clear and more phase II/III clinical trials on the antiviral effects of artemisinin and its derivatives are needed to promote the translation from laboratory to population. Artemisinin and its derivatives provided a model for the development of traditional medicine using modern science and technology. With the features of complex components and multiple targets and pathways, Traditional Chinese Medicine need extensive research on pharmacological and mechanism studies to maximize their clinical potentials [118–120].

### Supplementary Information

**Additional file 1: Table S1.** Description of the included studies by disease classification

## Data Availability

Not applicable.

## Abbreviations

CNKI: China National Knowledge Infrastructure
WHO: World Health Organization
LN: Lupus nephritis
VKH: Vogt-Koyanagi-Harada
RA: Rheumatoid arthritis
CIN: Cervical Intraepithelial Neoplasia
ACTs: Artemisinin-based combination therapy
COVID-19: Coronavirus Disease 2019
HIV: Human Immunodeficiency Virus
TACE: Transcatheter arterial chemoembolization
CMV: Cytomegalovirus
MTD: Maximum tolerated dose
ICD-11: The International Classification of Diseases, 11th Revision
