# Correction: New clinical application prospects of artemisinin and its derivatives: a scoping review

**DOI:** 10.1186/s40249-024-01180-w

**Published:** 2024-02-09

**Authors:** Yangmu Huang, Yang Yang, Guangqi Liu, Ming Xu

**Affiliations:** 1 School of Public Health, Peking University, 38 Xue Yuan Road, Haidian District, Beijing, 100191 China; 2 Institute for Global Health and Development, Peking University, 38 Xue Yuan Road, Haidian District, Beijing, 100191 China; 3 Energy Saving and Environmental Protection and Occupational Safety and Health Research Institute, China Academy of Railway Sciences Co., Ltd, No. 2 Daliushu Road, Beijing, 100081 China

**Correction** **:** **Infectious Diseases of Poverty (2023) 12:115** 10.1186/s40249-023-01152-6

Following publication of the original article [1], the authors reported that the below two corrections should be made in the original article.

1. The sentence “safe in pink, effective in blue and invalid in green” in figure legend of Fig. 3c should be updated to “safe in pink, effective in **green** and invalid in **yellow**”.

The full correct legend of Fig. 3 should read:

**a** Distribution of potential clinical applications of artemisinin and its derivatives. **b** Word cloud of research results on efficacy/safety for diseases (due to the excessive number of studies on schistosomiasis control, the image is not shown in this figure). The graph was based on the frequency of clinical studies of artemisinin and its derivatives in the treatment of different diseases. **c** Research status on the efficacy and safety of artemisinin and its derivatives for potential clinical applications. The upper semicircle represents the research results, and the lower semicircle represents the field of disease. Different colors are used to distinguish the research results, that is, safe in pink, effective in green and invalid in yellow. The width of the string is based on the number of studies that have corresponding results in this field.

2. Author Dan Hu is removed from the author list due to personal issues.

The correct Author Contribution section should read:

YH conceived the research proposal and guided the overall study. YY, GL, YH and MX screened the literature, extracted research information and resolved the disputes. YY and GL drafted the manuscript. MX and YH critically reviewed the manuscript. All authors read and approved the final manuscript.

The original article [1] has been updated.

## References

[1] Huang Y, Yang Y, Liu G, Xu M (2023). New clinical application prospects of artemisinin and its derivatives: a scoping review. Infect Dis Poverty.

