# Supplementary material for: New clinical application prospects of artemisinin and its derivatives: a scoping review
Source: Infect Dis Poverty. 2023 Dec 11;12:115. doi: 10.1186/s40249-023-01152-6 (PMC10712159; doi:10.1186/s40249-023-01152-6)
Supplement: Supplementary file 1 — Additional file 1: Table S1. Description of the included studies by disease classification [file 40249_2023_1152_MOESM1_ESM.docx]

**Table 3** Description of the inclusion study by disease classification

| Tittle | Author/Date | Classification | Disease | Country | Drug/Dose | Scale | AE | Result | Conclusion |
| --- | --- | --- | --- | --- | --- | --- | --- | --- | --- |
| Clinical observation of artesunate in the treatment of eczema-dermatitis and photosensitive dermatosis | Chen H et al,  1991 | Skin disease | Eczema,  Dermatitis, | China | Artesunate/ 60mg/d | 50 | 1 | Most of the patients were cured after 1-2 courses, and a few were cured after 3 courses, the effective time was 3 days, and the exudative lesions disappeared in about 1 week. | It was found that artesunate had a good long-term effect in the treatment of eczema and photosensitive dermatoses,  but poor in atopic dermatitis. |
| Clinical observation of artesunate in the treatment of rheumatoid arthritis | Cui XJ et al,  2007 | Inflammatory disease | Rheumatoid arthritis | China | Artesunate/ 200mg/d | 87 | 1 | Fifty cases of rheumatoid arthritis were treated with artesunate on the basis of prednisone tablets and methotrexate, all clinical indexes have been well recovered, which is similar to that of hydroxychloroquine. | The therapeutic effect and safety of artesunate on rheumatoid arthritis is no less than that of hydroxychloroquine. |
| Clinical observation of artemether in the treatment of pleomorphic sunrash and chronic actinic dermatitis | Dan-qi Deng et al,  2006 | Skin disease | Pleomorphic solar rash，Chronic actinic dermatitis | China | Artemether/ 80mg/ d | 168 | 1 | There was no significant difference in the total effective rate of pleomorphic solar eruption and chronic actinic dermatitis between the treatment group and the control group by chi-square test, and no side effects were found. | Artemether is effective, safe, convenient and economical in the treatment of PLE and CAD. It is valuable for further research, development and application. |
| Clinical Nursing of severe Lupus nephritis treated by combination of traditional Chinese and Western Medicine | Ai-hua Du et al,  2004 | Inflammatory disease | Lupus nephritis | China | Artesunate/100mg/d | 25 | 2 | Lupus nephritis was treated with hormone combined with cyclophosphamide combined with Ling dan tablet and artesunate. After treatment, complete remission was achieved in 8 cases and partial remission in 14 cases. The total effective rate was 84%. | Hormone combined with cyclophosphamide combined with Ling dan tablet and artesunate was used to treat lupus nephritis with satisfactory effect. |
| Clinical study of artemether combined with praziquantel in the treatment of acute schistosomiasis japonica | Hou XY et al,  2006 | Parasite | Schistosoma japonicum | China | Artemether/6mg/kg | 96 | 2 | Artemether 6mg/kg is safe, no obvious adverse reaction and good compliance in the treatment of acute schistosomiasis japonicum, and the cure rate and fecal egg reduction rate of acute schistosomiasis japonica treated with praziquantel combined are slightly higher than those of traditional praziquantel alone. | The combination of artemether and praziquantel is safe in the treatment of acute schistosomiasis, and there is no significant difference in the therapeutic effect between artemether and praziquantel alone. |
| Clinical observation of artesunate in preventing reinfection of Schistosoma japonicum | Hua HY  et al,  2010 | Parasite | Schistosoma japonicum | China | Artesunate/6 mg/kg | 176 | 1 | The phenomenon of anti-reinfection appeared in the medication group, and the effect of group I was more obvious. | Artesunate has a good effect on early treatment and prevention of schistosomiasis reinfection. The dose of 6mg/kg is better when it is taken once a week for 4 weeks. |
| Clinical study on the effect of artesunate on immune function in patients with lupus nephritis | HuangXX,  2011 | Inflammatory disease | Lupus nephritis | China | Artesunate/50mg/d | 60 | No side effects | Artesunate is similar to Tripterygium wilfordii polyglycoside tablets in total effective rate, improvement of LN symptoms and some laboratory indexes, but significantly superior to Tripterygium wilfordii polyglycosides tablets in total effective rate, improvement of systemic symptoms, reduction of activity index and immunological index. | Artesunate can comprehensively improve the articular systemic lesions of LN without obvious adverse reactions, so it is worth further popularizing and applying. |
| Observation on the efficacy of artesunate boric acid powder combined with microwave irradiation in the treatment of chronic suppurative otitis media in malaria area | Li DZ,  2015 | Inflammatory disease | Otitis media | [Cameroon](javascript:;) | Artesunate | 152 | without record | The total effective rate of the observation group (84%) was significantly better than that of the control group (57%), and the difference was statistically significant (P < 0.05). | Compared with traditional oral antibiotics and middle ear drops, artesunate borate powder can significantly improve the curative effect, shorten the course of disease, reduce recurrence and complications, and is worth popularizing in primary hospitals. |
| Clinical observation of oral artesunate in the treatment of 31 cases of rosacea | Li T  et al,  2015 | Skin disease | Rosacea | China | Artesunate/100mg/d | 65 | 1 | There was no significant difference in the effective rate and the incidence of adverse reactions between the treatment group and the control group. | The efficacy of artesunate tablets is similar to that of doxycycline hydrochloride enteric-coated capsules in the treatment of rosacea. |
| Clinical study of artesunate in the treatment of COVID-19 | Lin YR  et al,  2020 | Virus, Inflammatory disease | COVID-19 | China | Artesunate/120 mg/d | 43 | 3 | The symptom improvement time, 2019-NCOV nucleic acid test negative time, lung lesion absorption time and hospitalization time in artesunate combination treatment group were significantly shorter than those in conventional treatment group. There was no significant difference in the incidence of adverse drug reactions | Artesunate can shorten the treatment time of COVID-19 patients, improve prognosis and eliminate pathogens. It has few adverse reactions and has a good application prospect. |
| Clinical study of artesunate in the treatment of systemic lupus erythematosus | Liu HJ,  2002 | Inflammatory disease | Systemic lupus erythematosus | China | Artesunate/60mg/d,50mg/d | 149 | No side effects | The clinical symptoms and related laboratory indexes of the three groups were improved in varying degrees | This study is of great significance to broaden the scope of clinical application of artesunate (from the simple treatment of malaria to the treatment of autoimmune diseases). |
| Inhibitory effect of Cordyceps sinensis and artemisinin on recurrence of lupus nephritis | Lan Lu,  2002 | Inflammatory disease | Lupus nephritis | China | Artemisinin | 61 | without record | There were significant differences in markedly effective rate and creatinine clearance rate between the two groups. Cordyceps sinensis and artemisinin reduced the adverse reactions of the drug itself. | Cordyceps sinensis and artemisinin can inhibit the recurrence of lupus nephritis and protect renal function. |
| Effect of artesunate hepatic artery infusion on immune function in patients with primary liver cancer | Luo YC,  2008 | Tumor | Liver cancer | China | Artesunate/120mg/d | 60 | 2 | The treatment group was significantly better than the control group in improving immunity, and the two groups were similar in reducing tumor size, reducing the short-term efficacy of AFP and side effects of intervention | Artesunate combined with interventional therapy can improve the immunity of patients with primary liver cancer, but it increases the risk of liver function damage, which is of certain significance for the clinical anti-tumor study of artesunate. |
| Observation on the Clinical effect of Qing Hao and turtle shell decoction in the treatment of Hematological Diseases | Sun ZG,  2016 | Tumor | Hematological Diseases | China | Artemisinin | 16 | without record | In 5 cases of Hodgkin's lymphoma, fever decreased after 3 doses, and fever subsided after 7 doses. 4 cases of acute lymphoblastic leukemia with hyperhidrosis, body deficiency and hyperhidrosis were significantly improved after taking 3 doses. | The effect of Qing Hao and turtle shell decoction in the treatment of hematological diseases is satisfactory, which can effectively improve immunity and promote the rehabilitation of patients. |
| A comparative study of artemether in adjuvant treatment of schizophrenia with Toxoplasma gondii antibody positive | Wang DG  et al,  2012 | Parasite | Toxoplasma gondii | China | Artemether | 45 | 1 | There was no significant difference in the total scores of the clinical efficacy scale and the positive and negative symptom scale between the two groups at each period of treatment, but at the end of the 2nd, 4th and 6th week of treatment, the positive and negative symptom factor scores of the positive and negative symptom scale in the experimental group decreased more significantly than those in the control group. | Artemether can significantly improve some of the mental symptoms caused by Toxoplasma gondii infection: antipsychotic drugs combined with artemether therapy can effectively reduce the positive rate of Toxoplasma gondii antibody in patients with schizophrenia, and the safety is high. |
| Clinical study of artesunate/cisplatin-paclitaxel in the treatment of ovarian cancer | Wang M  et al,  2016 | Tumor | Ovarian cancer | China | Artesunate/ 1.2mg/g | 84 | 1 | The clinical treatment effect of observation group was higher than control group, and the occurrence of side effects and bone marrow suppression results were significantly lower than control group.  The above differences were statistically significant (P < 0.05) | Artesunate in the treatment of ovarian cancer can increase the sensitivity to cisplatin, improve its clinical effect, improve the quality of life of patients, and reduce adverse reactions. |
| Clinical observation of artesunate in the treatment of rheumatoid arthritis | Wei S,  2008 | Inflammatory disease | Rheumatoid arthritis | China | Artesunate/ 60mg/d | 40 | 2 | There was no statistical difference in clinical curative effect between the two groups, and there was no significant difference in each index between the two groups, and the improvement of symptoms and indexes was similar. There was no clinically significant abnormality between the two groups before and after treatment. | The results of clinical observation support the immunological basis of artesunate in the treatment of RA. |
| Observation on the efficacy of chemotherapy combined with sequential administration of artesunate in the treatment of non-small cell lung cancer | Xiao XP  et al,  2015 | Tumor | non-small cell lung cancer | China | Artesunate/ 1~8d ：2×120mg/d，after：3×80mg/d | 113 | without record | After the end of chemotherapy, there was no significant difference in the effective rate between the two groups, and the disease control of the experimental group was higher than that of the control group. The time of tumor progression in the test group was longer than that in the control group. The 1-year survival rate of the test group was higher than that of the control group | Chemotherapy combined with sequential administration of artesunate can improve the disease control rate, prolong the time of tumor progression and 1-year survival rate in patients with NSCLC after chemotherapy. |
| Clinical observation on prevention of schistosomiasis by oral artesunate in people exposed to epidemic water for a short time | Yi ZH et al,  2000 | Parasite | Schistosoma japonicum | China | Artesunate/6mg/kg | 680 | 1 | After taking Art, a few patients complained of dizziness, headache, fatigue, lethargy and other reactions. The other clinical indicators were within the normal range. The protective rate of artesunate against schistosomiasis japonicum was 89.18%. | This observation table Art adopts the prevention scheme of 6mg/kg/ × 3 times, which has good effect, little side effect, high safety, simple and economical, and has a good prospect of popularization and application when used in the prevention of schistosomiasis japonica in people exposed to epidemic water for a short time. |
| Clinical observation of artesunate in the treatment of 30 cases of systemic lupus erythematosus | Yu QB  et al,  1996 | Inflammatory disease | Systemic lupus erythematosus | China | Artesunate/adult：60mg/kg,child：1.2/kg | 30 | 1 | This paper reports the treatment of 30 cases of systemic lupus erythematosus with a total effective rate of 93.3%. | This study shows that artesunate has certain clinical value in the treatment of systemic lupus erythematosus. |
| Clinical analysis of 90 cases of dermatosis treated with artesunate | Yu QB  et al,  1997 | Skin disease | Eczema, Atopic dermatitis, Pleomorphic erythema, Polymorphic solar rash, Summer blister disease, soriasis vulgaris, Dermatomyositis | China | Artesunate/ adult：60mg/kg,child：1.2/kg | 90 | 1 | The effective rate for eczema, pleomorphic erythema, pleomorphic solar eruption and summer blister disease was 100%, and the effective rate for psoriasis vulgaris and dermatomyositis was 60% and 75%, respectively. | It is further demonstrated that artesunate has the effect of inhibiting allergic dermatitis and anti-photosensitivity, and has clinical value. |
| Therapeutic effect of artesunate on acute leukemia and its effect on intracellular calcium concentration | XJ Zhang,  2008 | Tumor | Leukemia | China | Artesunate/2×60mg /d | 20 | No side effects | The complete remission rate of the observation group was 60%, and the total effective rate was 80%; the complete remission rate of the control group was 40%, and the total effective rate was 50%. There was no significant difference between the two groups, and artesunate was safe and effective. | Both clinical and experimental aspects have confirmed that artesunate has obvious therapeutic effect on acute leukemia, indicating that artesunate is a safe and effective new anti-leukemia drug, which is worthy of clinical use. |
| Clinical efficacy and safety of artesunate in the treatment of advanced retinoblastoma | Yuan-yuan Zhang,  2015 | Tumor | Retinoblastoma | China | Artesunate/10mg/kg | 11 | 2 | Artesunate is effective and safe in the clinical treatment of advanced retinoblastoma. | The preliminary results show that artesunate is effective and safe in the clinical treatment of advanced retinoblastoma. |
| Clinical observation of artemether capsule combined with glucocorticoid in the treatment of 25 cases of VKH syndrome | Zhao YJ,  2016 | Inflammatory disease | Uveitis | China | Artemether/80mg/d | 25 | 1 | Artemether capsule combined with glucocorticoid can effectively control uveal inflammation, reduce ophthalmopathy complications, improve corrected visual acuity and prolong recurrence time. | Artemether capsule combined with glucocorticoid is safe and effective in the treatment of VKH syndrome. |
| Safety of artesunate in the treatment of systemic lupus erythematosus | Jia-xi Zhong et al,  2000 | Inflammatory disease | Systemic lupus erythematosus | China | Artesunate/ 2 mg/kg/d | 32 | 1 | Safety observation of artesunate in the treatment of systemic lupus erythematosus | Artesunate was used to treat 34 cases of systemic lupus erythematosus. The safety of artesunate was observed (including blood routine, liver and kidney function and electrocardiogram). No obvious side effects were found. |
| Study on the clinical efficacy of anti-tumor angiogenesis drugs | Wen-jin Zhou et al,  2017 | Tumor | Lung cancer, Stomach cancer, Breast cancer, Liver cancer, Esophageal cancer | China | Artesunate | 132 | without record | The effective rate of artesunate is 85%. | Artesunate has a good clinical effect on tumor angiogenesis |
| Cardiac safety of artemisinin-based combination therapy among adults infected with human immunodeficiency virus and stabilized on antiretroviral therapy in Malawi | C.G.Banda et al,  2015 | Virus | HIV | [Malawi](javascript:;) | Dihydroartemisinin, Artemether, Artesunate. | 154 | without record | Artemisinin-based combination therapies have a strong safety profile in people with malaria-HIV co-infection | The strong safety of artemisinin-based combination therapies in people with malaria-HIV co-infection will help provide treatment guidelines |
| A phase I study of intravenous artesunate (IV AS) in patients with solid tumors | J.F.Deeken et al,  2015 | Tumor | Solid tumor | [America](javascript:;) | Artemisinin/ 8、12、18、25、34、45 mg/kg | 19 | without record | The MTD of IV AS is 18mg/kg on this schedule. Treatment was well tolerated at that dose level. Modest clinical activity was seen | The MTD of IV AS is 18mg/kg on this schedule. Treatment was well tolerated at that dose level. Modest clinical activity was seen |
| Antischistosomal efficacy of artesunate combination therapies administered as curative treatments for malaria attacks | B. Denis,  2007 | Parasite | Schistosoma japonicum | [Senegal](javascript:;) | Artesunate/4mg/kg/d | 27 | [without](javascript:;) [record](javascript:;) | The overall cure rate and reduction in the mean number of excreted eggs at 28 days post treatment were 92.6% and 94.5%, respectively. | Our findings indicate that artesunate, in addition to being a very effective treatment for uncomplicated malaria, can also sharply reduce the S. haematobium loads harboured by pre-school African children |
| Preventive effect of artemether on schistosome infection | Y. Song et al,  1998 | Parasite | Schistosoma japonicum | China | Artemether/6mg/kg | 209 | No side effects | In Art group, 99 individuals receiving 3 doses of the drug completed the stool examination with egg-positive rate of 4% and no acute schistosomiasis was seen. In the control group, among 110 people who completed the observation, 44 were egg-positive with an infection rate of 40%, and 29 were identified as having acute schistosomiasis. | After oral Art was given to the people fighting against flood in schistosomiasis endemic area of Poyang Lake, it was shown that the oral Art has a promising effect on controlling acute schistosomiasis and reducing the infection rate. |
| A first-in-human proof-of-concept trial of intravaginal artesunate to treat cervical intraepithelial neoplasia (CIN2/3) | C.L. Trimble et al,  2020 | Tumor | Cervical intraepithelial neoplasia | [America](javascript:;) | Artesunate/50、200mg | 28 | 2 | Reported adverse events were mild and self-limited. In the modified intention-to-treat analysis, histologic regression was observed in 19/28 (67.9 %) subjects. Clearance of HPV genotypes detected at baseline occurred in 9 of the 19 (47.4%) subjects whose lesions underwent histologic regression. | Self-administered intravaginal artesunate inserts were safe and well-tolerated, at clinically effective doses to treat CIN2/3. These findings support proceeding with phase II clinical studies. |
| Field studies on the preventive effect of oral artemether against schistosomal infection | S. Xiao et al,  1996 | Parasite | Schistosoma japonicum | China | Artemether/ 6mg/kg | 741 | No side effects | In the Art group, an infection rate of 5.5%, while in the control group, an infection rate of 13.6%. The egg count per gram of feces (EPG) determined was 122 ± 79 in the Art group and 681 ± 909 in the control group. No apparent adverse side effect was seen with Art | Oral Art exhibited apparent preventive effect on the residents who contacted the infested water in schistosomiasis endemic area. |
| Artesunate combined with vinorelbine plus cisplatin in treatment of advanced non-small cell lung cancer: A randomized controlled trial | S. q. Yu et al,  2008 | Tumor | non-small cell lung cancer | China | Artesunate/ 120 mg/d | 120 | No side effects | There were no significant differences in the short-term survival rate, MST and 1-year survival rate between the trial group and the control group, which were 45.1% and 34.5%, 44 weeks and 45 weeks, 45.1% and 32.7%, respectively (P > 0.05). The DCR of the trial group (88.2%) was significantly higher than that of the control group (72.7%) | Artesunate can be used in the treatment of NSCLC. Artesunate combined with NP can elevate the short-term survival rate and prolong the TTP of patients with advanced NSCLC without extra side effects. |
| A randomized, double-blind, placebo-controlled trial of safety and efficacy of combined praziquantel and artemether treatment for acute schistosomiasis japonica in China | Hou XY  et al,  2008 | Parasite | Schistosoma japonicum | China | Artemether/ 60 mg/kg | 205 | 1 | Treatment efficacies of the four different treatment regimens were 98.0%, 96.4%, 97.7% and 95.7% for group A, B, C, and D respectively (P > 0.05). The group B had a greater treatment efficacy (96.4%) than the group D (95.7%) (P > 0.05). Group A treatment was better for clearance of fever (P < 0.05) and resulted in a shorter hospitalization time (P < 0.05). | The combination of AM and PZQ chemotherapy did not improve treatment efficacy compared with PZQ alone. |
| Use of a physiologically-based pharmacokinetic model to simulate artemether dose adjustment for overcoming the drug-drug interaction with efavirenz | Marco Siccardil  et al,  2013 | Virus | HIV | [Britain](javascript:;) | Artemether/80mg/d | 50 | without record | Dose increases of artemether, to correct for the drug-drug interaction, were simulated and a dose of 240 mg was predicted to be sufficient to overcome the interaction and allow therapeutic plasma concentrations of artemether. | Pharmacokinetics and drug-drug interactions are consistent with available clinical data. |
| Evaluation of the efficacy and tolerance of artemether emulsion for the treatment of papulopustular rosacea: A randomized pilot study | Guo-jiang Wang et al,  2019 | Skin disease | Rosacea | China | Artemether/1% | 130 | 1 | Artemether emulsion improved papulopustular rosacea in the metronidazole emulsion group as early as 4 weeks, but its beneficial effect was maintained through the 8-week follow-up period compared to metronidazole emulsion. | Artemether emulsion improved papulopustular rosacea in the  metronidazole emulsion group as early as 4 weeks, but its beneficial effect was maintained through the 8-week follow-up period compared to metronidazole emulsion. |
| Efficacy and safety of artemether emulsion for the treatment of mild-to-moderate acne vulgaris: A randomized pilot study | Wen-tong Shen et al,  2021 | Skin disease | Acne vulgaris | China | Artemether/1% | 73 | 1 | After 12 weeks, patients randomized to the artemether emulsion group received artemether emulsion had significantly lower GAGS scores compared to patients who received fusidic acid emulsion. No major adverse events were noted in either treatment group through 12 weeks. | Artemether emulsion had better effect in improving mild-to-moderate AV compared to fusidic acid emulsion with barely AEs. |
| A double-blind field trial on the effects of artemether on Schistosoma japonicum infection in a highly endemic focus in southern China | Yue-Sheng Li et al,  2005 | Parasite | Schistosoma japonicum | China | Artemether/ 6 mg/kg | 783 | without record | Compared to the baseline, the geometric mean intensity of S. japonicum infection had decreased by 96.1% in the artemether group, and increased by 50.8% in the placebo group. No acute cases of schistosomiasis japonica were observed in the artemether group, whereas three such cases were reported from the placebo group. | This study confirms that repeated oral artemether produces no drug-related adverse effects, significantly reduces  incidence and intensity of patent S. japonicum |
| Prophylactic effect of artemether on human schistosomiasis mansoni among Egyptian children: A randomized controlled trial | Hala Elmorshedy et al,  2016 | Parasite | Schistosoma japonicum | [Egypt](javascript:;) | Artemether/6mg/kg | 913 | without record | prevalence of infection among the PZQ/ART was approximately half that of the PZQ/ART-placebo group, i.e. 6.7% versus 11.6%, and incidence of new infections for the PZQ/ART was 2.7% versus 6.5% for the PZQ/ART-placebo. | PZQ/ART combined therapy might be considered as an adjunct measure against human schistosomiasis, by specifically reducing transmission and therefore contribute to disease elimination. |
| Artesunate plus sulfamethoxy pyrazine/pyrimethamine for the treatment of cutaneous leishmaniasis: a double-blind, placebo-controlled clinical trial | Ayoub A. Mohamed et al,  2009 | Skin disease | Cutaneous leishmaniasis | [Sudan](javascript:;) | Artesunate/100mg/d | 640 | No side effects | The efficacy and safety of oral artesunate+ sulfadoxine / pyrimethamine (AS+SP) (4mg/kg AS for 3 consecutive days+25mg sulfadoxine on Day 0) in the treatment of Schistosoma mansoni infections were compared with those of praziquantel (PZQ) (40 mg/kg) | AS+SP has poor efficacy in the treatment of S. mansoni compared with PZQ. |
| An artesunate-containing antimalarial treatment regimen did not suppress cytomegalovirus viremia | Soren Gantt et al,  2013 | Virus | CMV | [Uganda](javascript:;) | Artesunate/4 mg/kg/d | 493 | without record | CMV was detected in 11.4% of children immediately prior to treatment and 10.7% 3 days later (P = 0.70). The average quantity of CMV was 0.30 log10copies per million cells higher on day 3 than at treatment initiation (95% CI 0.01–0.58, P = 0.041) | A standard 3-day artesunate-containing antimalarial regimen had no detectable effect on CMV viremia in children with malaria. Longer treatment courses and/or higher doses of artesunate than those routinely used for malaria may be required for effective treatment of CMV infection. |
| The effect of artemether on psychotic symptoms and cognitive impairment in first-episode, antipsychotic drug-naive persons with schizophrenia seropositive to Toxoplasma gondii | Hui-Ling Wang et al,  2014 | Parasite | Toxoplasma gondii | China | Artemether/80 mg | 100 | 1 | There were also no significant differences between the two groups in performance on any of the Brief Assessment of Cognition in Schizophrenia (BACS) cognitive domains. | The artemether-risperidone combination is safe and well tolerated, but artemether as an adjunct to risperidone does not appear to alleviate cognitive deficits of schizophrenia. |
| Artemisinin reduces the level of antibodies to gliadin in schizophrenia | Faith Dickerson et al,  2011 | Parasite | Toxoplasma gondii | [America](javascript:;) | Artemisinin/100mg/d | 66 | No side effects | The medication was well tolerated and there were no significant side effects associated with the treatment regimen. There was no significant difference in the change of positive, negative, general, or total PANSS symptoms between groups. | The study did not demonstrate clinical benefit of adjunctive artemisinin for schizophrenia symptoms. The finding of reduced levels of antibodies to gliadin in the artemisinin group merits further study. |
| Efficacy of a combination of praziquantel and artesunate in the treatment of urinary schistosomiasis in Nigeria | P.C.Inyang-Etoh et al,  2008 | Parasite | Schistosoma japonicum | Nigeria | Artesunate/ 40mg/kg | 327 | No side effects | All treatment regimens were well tolerated. The cure rates were 72.7% in the praziquantel plus placebo-treated group and 70.5% in the artesunate plus placebo group, while the artesunate plus praziquantel group had the highest cure rate (88.6%). | This study confirmed that the treatment of urinary schistosomiasis with the combination of praziquantel and artesunate is safe and more effective than treatment with either drug alone. |
| Praziquantel, Mefloquine-Praziquantel, and Mefloquine-Artesunate-Praziquantel against Schistosoma haematobium: A Randomized, Exploratory, Open-Label Trial | Jennifer Keiser et al,  2014 | Parasite | Schistosoma japonicum | Côte d'Ivoire | Artesunate/ 100 mg/d | 61 | 1 | No difference in efficacy was observed between the three treatment groups on either follow-up. Praziquantel monotherapy was the best tolerated treatment. With the exception of abdominal pain at moderate severity, adverse events were mild. | The addition of mefloquine or mefloquine artesunate does not increase the efficacy of praziquantel against chronic S. haematobium infection. Additional studies are necessary to elucidate the effect of the combinations against acute schistosomiasis. |
| Lower artemether, dihydroartemisinin and lumefantrine concentrations during rifampicin-based tuberculosis treatment | Mohammed Lamordea et al,  2012 | Inflammatory disease | Tuberculosis | Uganda | Artemether/80mg/d | 11 | without record | Artemether and dihydro-artemisinin Cmax were 83% (0.17,0.08–0.39) and 78% (0.22, 0.15–0.33) lower, respectively, during rifampicin treatment. | Pharmacokinetic parameters for artemether lumefantrine were markedly lower during rifampicin-based tuberculosis treatment. Artemether lumefantrine should not be co-administered with rifampicin. |
| Efficacy of artesunate in the treatment of urinary schistosomiasis, in an endemic community in Nigeria | P.C.Inyangetoh et al,  2004 | Parasite | Schistosoma japonicum | Nigeria | Artesunate/ 6 mg/kg | 500 | 1 | The artesunate was well tolerated. In the Adim community at least, it would be more cost-effective to treat urinary schistosomiasis with artesunate than with praziquantel. | The wide-spread use of artesunate against schistosomiasis has to be considered carefully, however, if it is not to compromise the efficacy of the drug as an antimalarial, by increasing the risk of resistance developing in local Plasmodium. |
| Efficacy of artesunate and praziquantel in Schistosoma Haematobium infected schoolchildren | D.DeClercq et al,  2001 | Parasite | Schistosoma japonicum | [Senegal](javascript:;) | Artesunate/50mg/d | 288 | 1 | high and nearly comparable egg count reduction rates were obtained with both drugs at each follow-up after treatment (5, 12 and 24 weeks) in the heavy infected group of children (>50 eggs/10 ml of urine). No major adverse effects were observed. | Artemisinin and its derivatives, widely used for the treatment of malaria, also display antischisto-somal properties. |
| Efficacy of artesunate with sulfalene plus pyrimethamine versus praziquantel for treatment of Schistosoma mansoni in Kenyan children: an open-label randomised controlled trial | Charles O Obonyo et al,  2010 | Parasite | Schistosoma japonicum | [Kenya](javascript:;) | Artesunate/ 100 mg/d | 212 | 1 | 69 patients (65%) were cured in the praziquantel treatment group compared with 15 (14%) in the artesunate with sulfalene plus pyrimethamine treatment group (p<0·0001). children treated with artesunate with sulfalene plus pyrimethamine had fewer adverse events than did those treated with praziquantel | The standard treatment with praziquantel is more effective than artesunate with sulfalene plus pyrimethamine in the treatment of children with S mansoni infection in western Kenya. Whether artemisinin-based combination therapy has a role in the treatment of schistosomiasis is unclear. |
| Efficacy of Artesunate + Sulfamethoxypyrazine/Pyrimethamine versus Praziquantel in the Treatment of Schistosoma haematobium in Children | Mahamadou S. Sissoko et al,  2009 | Parasite | Schistosoma japonicum | Bamako | Artesunate/300mg | 800 | 1 | The study demonstrates that PZQ was more effective than AS+SMP for treating Schistosoma haematobium. However, the safety and tolerability profile of AS+SMP was similar to that seen with PZQ | further investigations seem justifiable to determine the dose/efficacy/safety pattern of AS+SMP in the treatment of Schistosoma infections. |
| Prospective open uncontrolled phase I study to define a well tolerated dose of oral artesunate as add-on therapy in patients with metastatic breast cancer (ARTIC M33/2) | Cornelia von Hagens et al,  2017 | Tumor | breast cancer | [Germany](javascript:;) | Artesunate/ 100，150， 200 mg | 23 | without record | During the actual trial period of 4 ± 1 weeks, three patients experienced six DL-AEs altogether (leucopenia, neutropenia, asthenia, anemia) possibly related to ART (not exceeding 33% in any dose level). | Up to 200 mg/d (2.2–3.9 mg/kg/d) oral ART were safe and well tolerated; therefore, 200 mg/d are recommended for phase II/III trials |
| The interaction between artemether lumefantrine and lopinavir/ritonavir-based antiretroviral therapy in HIV-1 infected patients | T. Kredo et al,  2016 | Virus | HIV | [South](javascript:;) [Africa](javascript:;) | Artemether/ 80 mg | 34 | 1 | There were no serious adverse events and no difference in electrocardiographic QTcF- and PR-intervals, at the predicted lumefantrine Tmax. | Despite substantially higher lumefantrine exposure, intensive monitoring in our relatively small study raised no safety concerns in HIV-infected patients stable on lopinavir-based antiretroviral therapy given the recommended artemether-lumefantrine dosage. |
| Efficacy and safety of praziquantel and dihydroartemisinin piperaquine combination for treatment and control of intestinal schistosomiasis: A randomized, noninferiority clinical trial | Rajabu Hussein Mnkugwe et al,  2020 | Parasite | Schistosoma japonicum | [Tanzania](javascript:;) | Dihydroartemisinin/40mg | 639 | 1 | Praziquantel and Dihydroartemisinin piperaquine combination therapy is safe, and more efficacious compared to praziquantel alone for the treatment of intestinal schistosomiasis. | Further studies are needed to explore if the combination therapy can be considered as an option for mass drug administration to control and eventually eliminate schistosomiasis. |
| Population pharmacokinetics of artesunate and dihydroartemisinin during long-term oral administration of artesunate to patients with metastatic breast cancer | Therese Ericsson et al,  2014 | Tumor | Breast cancer | [Germany](javascript:;) | Dihydroartemisinin/ 100，150，200 mg/d | 23 | without record | A time-dependent increase in apparent elimination clearance of DHA was observed. | Population pharmacokinetics of ARS and DHA in patients with breast cancer was well described by a combined drug-metabolite model without any covariates and with an increase in apparent elimination clearance of DHA over time. |
| Artesunate and Praziquantel for the Treatment of Schistosoma haematobium Infections: A Double-Blind, Randomized, Placebo-Controlled Study | Steffen Borrmann et al,  2001 | Parasite | Schistosoma haematobium | [Gabon](javascript:;) | Artesunate/4mg/kg/d | 300 | 1 | All treatment regimens were well tolerated. The praziquantel plus placebo - treated group attained a cure rate of 73%, artesunate plus placebo a rate of 27%, the combination of artesunate and praziquantel a rate of 81%, and placebo alone a rate of 20% | In summary, earlier findings of efficacy of artemisinin derivitives against S. mansoni and S. japonicum could not be confirmed in S. haematobium infections. |
| A Randomized Controlled Pilot Study of Artesunate versus Triclabendazole for Human Fascioliasis in Central Vietnam | Tran Tinh Hien et al,  2008 | Parasite | Human Fascioliasis | Vietnam | Artesunate/4 mg / kg/d | 100 | without record | Patients treated with artesunate were significantly more likely to be free of abdominal pain at hospital discharge, but the complete response rate at 3 months was lower than for patients treated with triclabendazole | There may be a role for artesunate in fascioliasis |
| Lopinavir/ritonavir significantly influences pharmacokinetic exposure of artemether/ lumefantrine in HIV-infected Ugandan adults | Pauline Byakika  Kibwika et al,  2012 | Virus | HIV | [Uganda](javascript:;) | Artesunate,  Dihydroartemisinin/80 mg | 29 | No side effects | Co-administration of artemether/ lumefantrine with lopinavir/ritonavir significantly reduced artemether maximum concentration (Cmax) and area under the concentration-time curve (AUC). Dihydroartemisinin Cmax and AUC were not affected | Co-administration of artemether / lumefantrine with lopinavir/ritonavir significantly increases lumefantrine exposure, but decreases artemether exposure. |
| Interaction between Artemether-Lumefantrine and Nevirapine-Based Antiretroviral Therapy in HIV-1-Infected Patients | T. Kredo et al,  2011 | Virus | HIV | [South](javascript:;) [Africa](javascript:;) | Artemether/ 80 mg | 36 | 1 | Nevirapine-based ART decreased artemether and dihydroartemisinin AUCs but unexpectedly increased lumefantrine exposure. | The mechanism of the lumefantrine interaction remains to be elucidated. Studies investigating the interaction of nevirapine and artemether-lumefantrine in HIV-infected patients with malaria are urgently needed. |
| The sensitivity of artesunate against Schistosoma japonicum decreased after 10 years of use in China | Hai-Yong Hua et al,  2010 | Parasite | Schistosoma japonicum | China | Artesunate/ 6 mg/kg/300mg (≥50kg) | 216 | without record | In the current study, we conducted a doubleblind trial and found that the protection rate of artesunate was only 13.5% in the Administration I group | The sensitivity of S. japonicum to artesunate was confirmed to have decreased after being using for over 10 years. |
| A Randomised, Double Blind, Placebo-Controlled Pilot Study of Oral Artesunate Therapy for Colorectal Cancer | Sanjeev Krishna et al,  2015 | Tumor | Colorectal Cancer | [Britain](javascript:;) | Artesunate/ 200  mg | 23 | 1 | The primary outcome measure was the proportion of tumor cells undergoing apoptosis (significant if >. 7% showed Tunel staining). Apoptosis in >. 7% of cells was seen in 67% and 55% of patients in artesunate and placebo groups, respectively. | Artesunate has anti-proliferative properties in CRC and is generally well tolerated. |
| Efficacy and safety of mefloquine, artesunate, mefloquine-artesunate, tribendimidine, and praziquantel in patients with Opisthorchis viverrini: A randomised, exploratory, open-label, phase 2 trial | Soukhathammavong, P  et al,  2011 | Parasite | Opisthorchis viverrini | Laos | [Artesunate](javascript:;)/ 10mg/kg | 125 | 1 | Most adverse events were mild or moderate and affected all treatment groups; serious adverse events-vertigo, nausea, vomiting, and anxiety-were reported only by patients taking mefloquine or mefloquine-artesunate. | Mefloquine, artesunate, and mefloquine-artesunate did not show an effect. |
| Artesunate plus sulfadoxine/pyrimethamine versus praziquantel in the treatment of Schistosoma mansoni in eastern Sudan | Mohamed, A. A  et al,  2009 | Parasite | Schistosoma  mansoni | Sudan | [Artesunate](javascript:;)/4mg/kg | 92 | 1 | The cure rate at 28 days was 58.6% in the AS+SP group and 100% in the PZQ group (P < 0.001). Drug-related adverse effects (headache, dizziness, nausea and diarrhoea) were not significantly different between the two groups. | AS+SP has poor efficacy in the treatment of S. mansoni compared with PZQ |
| Efficacy and Safety of Artemether in the Treatment of Chronic Fascioliasis in Egypt: Exploratory Phase-2 Trials | Jennifer Keiser et al,  2011 | Parasite | Human Fascioliasis | [Egypt](javascript:;) | Artemether/6×80 mg and 3×200 mg | 36 | 1 | CRs achieved with 6×80 mg and 3×200 mg artemether were 35% and 6%, respectively. Artemether was well tolerated. A high efficacy was observed with triclabendazole administered at 10 mg/kg (16 patients; CR: 67%, ERR: 94%) and 20 mg/kg (4 patients; CR: 75%, ERR: 96%). | Artemether, administered at malaria treatment regimens, shows no or only little effect against fascioliasis, and hence does not represent an alternative to triclabendazole. The role of artemether and other artemisinin derivatives as partner drug in combination chemotherapy remains to be elucidated. |
| Field study of long-term oral artesunate in the prevention of schistosomiasis japonica | Liu HY  et al,  1999 | Parasite | Schistosoma japonicum | China | [artesunate](javascript:;)/ 6mg／kg 300mg(≥50kg) | 678 | 1 | In the heavily endemic area of schistosomiasis, residents took oral artesunate 6mg/kg once a week for 8 times during the infection season. The prevention and protection rate was 100%, and the side effect rate was significantly higher than that of the control group. | In the infection season of heavy epidemic areas of schistosomiasis, long-term oral artesunate is effective, safe and low toxic in the prevention of schistosomiasis. |
| Field study of oral artesunate in the prevention of schistosomiasis japonica | Ming-sheng Xu  et al,  1998 | Parasite | Schistosoma japonicum | China | [Artesunate](javascript:;)/ 6mg／kg 300mg(≥50kg) | 910 | 1 | The results showed that the protective rates of artesunate against schistosomiasis japonica were 80.94% and 100%, respectively. | The results showed that oral artesunate tablets had a good effect on the prevention of Schistosoma japonicum infection in the peak season of infection in beach and lake-marsh areas. |
| Experimental study on oral artesunate in the prevention of schistosomiasis japonica | Liu ZD  1994 | Parasite | Schistosoma japonicum | China | [Artesunate](javascript:;)6mg/kg | 500 | 1 | No case was positive in stool test, but the positive rate in the control group was 14.5% | The drug is used in pre-prevention to achieve 100% protection rate, easy to take, safe to use, and reliable in effect. |
| Study on the optimal scheme of oral artesunate to prevent Schistosoma japonicum infection | Lu GY  et al,  2000 | Parasite | Schistosomiasis | China | [Artesunate](javascript:;)/ 6 mg/kg | 1462 | 1 | The prevention and protection rates of people exposed to epidemic water in the short, medium and long term were 100%, 100% and 94.48%, respectively. | The results of this study showed that different administration regimens for different susceptible populations could effectively prevent and protect schistosomiasis, and the protective rate was 94. 48% ~ 100%, and the side effects were slight. |
| Field observation on prevention of Schistosoma japonicum infection by oral administration of artesunate at intervals of 15 days | Dan-dan Lin  et al,  1999 | Parasite | Schistosoma japonicum | China | [Artesunate](javascript:;)/50mg/kg | 786 | 1 | The positive rate of feces test was 2.65%.  The positive rate of fecal test in placebo group was 8.33% | On the basis of 100% protection rate of artesunate for 7 days, the same dose of artesunate for 15 days could still prevent schistosoma japonicum infection with minor side effects, which provided scientific basis for popularization and application of artesunate in epidemic areas and optimization of schistosomiasis control strategy. |
| Clinical verificati-on report of artesunate in the prevention of schistosomiasis japonica in Jishan | Zhi-de  Liu  et al,  1996 | Parasite | Schistosoma japonicum | China | [Artesunate](javascript:;)/ 6mg／kg 300mg(≥50kg) | 400 | 1 | 185 cases in the experimental group were negative, while 166 cases in the control group, the positive rate of class test was 4.22%. | The results showed that artesunate could be used to prevent schistosomiasis japonica |
| Clinical trial of artesunate in the prevention of schistosomiasis japonica | Shao-ji Zhang  et al,  2000 | Parasite | Schistosoma japonicum | China | [Artesunate](javascript:;)/ 6mg/kg | 3461 | 1 | Among the 5 pilot villages, the protection rate of experimental population was 100.0% in 4 villages and 89.1% in 1 village | The seven-day interval regimen, in which artesunate 6mg/kg‚ is taken seven days after contact with the infected water, works best |
| Randomized, double-blind, placebo-controlled trial of oral artemether for the prevention of patent Schistosoma haematobium infections | Hou XY  2007 | Parasite | Schistosoma japonicum | China | Artemether/ 6mg/kg | 205 | 2 | The cure rate and worm reduction rate of the four treatment regimens were all above 95%, and the treatment effect was good. | Artemisia annua 6mg/kg single dose in the treatment of acute schistosomiasis is safe, and the patient compliance is good. Artemisylaldehyde combined with praziquantel has no contrain-dications, and the cure rate is slightly higher than that of traditional single drug, but there is no significant difference in therapeutic effect. |
| Randomized, double-blind, placebocontrolled trial of oral artemether for the prevention of patent Schistosoma haematobium infections | [Eliézer K N'Goran](https://pubmed.ncbi.nlm.nih.gov/?term=N%27Goran+EK&cauthor_id=12556143)  et al,  2000 | Parasite | Schistosomiasis | [Cote](javascript:;) [d'Ivoire](javascript:;) | Artemether/6mg/kg | 440 | 1 | The incidence of patent S. haematobium infections in artemether recipients was significantly lower than in placebo recipients (49% versus 65%, protective efficacy: 0.25, 95% CI: 0.08-0.38, P = 0.007). | previous findings of efficacy of artemether against S. japonicum and S. mansoni were confirmed for S. haematobium, although the protective efficacy was considerably lower. |
| Clinical validation of artesunate against Schistosomiasis japonicum | Ren-gui Lu  et al,  1997 | Parasite | Schistosoma japonicum | China | [Artesunate](javascript:;)// 6mg／kg 300mg(≥50kg) | 208 | 1 | 56 patients in artesunate group were negative by Kato's method and incubation method 4 weeks after stopping taking artesunate. Among the 52 cases in the placebo group, 8 cases were positive for 15.38%. | The protective rate of artesunate against schistosomiasis japonicum was 100%, and the drug was safe and easy to take. |
| A phase I study of intravenous artesunate in patients with advanced solid tumor malignancies | Deeken, J. F  et al,  2018 | Tumor | Advanced solid tumor malignancies | America | [Artesunate](javascript:;)/ 8、12、18、25、34 and 45 mg/kg | 19 | without record | The MTD was determined to be 18 mg/kg. No responses were observed, | The MTD of intravenous artesunate is 18 mg/kg on this schedule. Treatment was well tolerated. |
| Investigation of ototoxicity of artesunate as add-on therapy in patients with metastatic or locally advanced breast cancer: new audiological results from a prospective, open, uncontrolled, monocentric phase I study | König, M.  et al,  2016 | Tumor | Breast cancer | [Germany](javascript:;) | [Artesunate](javascript:;)/100、150、200 mg | 23 | 1 | During the trial phase, four patients experienced auditory adverse events (AEs), possibly related to the ingestion of ART. Four patients developed vestibular system (vertigo)AEs during the trial phase, including one with SAE. SAE was completely reversible after termination of ART therapy. | None of the audiological results after 4 weeks of therapy with ART showed any dose-limiting auditory toxicity |
| Long-term add-on therapy (compassionate use) with oral artesunate in patients with metastatic breast cancer after participating in a phase I study (ARTIC M33/2) | von Hagens, C.  et al,  2019 | Tumor | Breast cancer | [Germany](javascript:;) | [Artesunate](javascript:;)/ 100、150、 200 mg | 25 | without record | A total of 25 AEs grade ≥ 2 at least possibly related to ART long-term add-on therapy were documented, two, six and 17 in dose groups 100, 150 and 200 mg/d ART respectively. Six of these AEs were classified as grade 3, two in patients taking 150 and four in patients on 200 mg/d, none of them being probably or certainly related to ART. | In thirteen patients with metastatic breast cancer up to 200 mg/d long-term oral ART (2.3-4.1 mg/kg BW/d) in up to 1115 cumulative treatment days (37 months) did not result in any major safety concerns. |
| Safety and efficacy of artemisinin-piperaquine for treatment of COVID-19: an open-label, non-randomised and controlled trial | G. Li, M.  et al,  2021 | Virus | COVID-19 | China | Artemisinin/125 mg(1d), 62.5 mg/d | 41 | 2 | The mean time to reach undetectable viral RNA (mean ± standard deviation) was 10.6 ± 1.1 days for the AP group and 19.3 ± 2.1 days for the control group. The CT imaging within 10 days post-treatment showed no significant between-group differences (P > 0.05). Both groups had mild adverse events. | For patients with mild to moderate COVID-19, patients in isolation and during isolation, and patients in close contact with COVID-19, the use of 8 tablets of AP over 7 days is recommended, especially in areas lacking medical facilities. |
